# EAN Perspective

**DOI:** 10.1192/j.eurpsy.2021.41

**Published:** 2021-08-13

**Authors:** C. Bassetti

**Affiliations:** Department of Neurology, Insel Gruppe AG, Inselspital, Bern, Switzerland

## Abstract

29th European Congress of Psychiatry (EPA 2021) „You can tell a good workman by his tools: The instruments of psychiatrists, psychologists and neurologists: Why so different?“ The term psychology („the study of the soul“) appeared for the first time in a printed book of Freigius in 1578, while the term neurology („the study of the form and function of the nervous system“) was coined by Willis in 1664 and that of psychiatry („the medical treatment of the soul“) by Reil in 1808. First physicians to devote entirely to neurology appeared in the midst of the 19th century in France, Germany, and England. Around this time neurology, (biological) psychiatry and (experimental) psychology converged to share similar roots in the brain. The three disciplines separated (again) at the beginning of the 20th century. Neurology remained for over 100 years mainly a diagnostic discipline, in which history and clinical examination were expected to lead to the identification of a topographic syndrome (or lesion) and eventually its etiology. In the last 30 years neurology underwent a revolution. While the importance (and validity) of phenotypical diagnoses remained, new (e.g. neuroimaging, genetic) tools have made precise diagnoses and causal treatments increasingly possible, transforming neurology into a treating discipline. The author will discuss why the separation between neurology, psychiatry and psychology is artificial (and even harmful for patients), how the multidimensional tools developed over the years by these disciplines can be of common interest, and what the EAN does to promote interdisciplinary scientific, educational, and political collaborations.

**Disclosure:**

No significant relationships.

